# “AACHEN” e-Learning Tool in Augmentative and Alternative Communication for Medical Students in Germany: Cross-Sectional Evaluation Study

**DOI:** 10.2196/88173

**Published:** 2026-04-29

**Authors:** Jessica Büchs, Christiane Neuschaefer-Rube

**Affiliations:** 1 Clinic for Otorhinolaryngology, Phoniatrics and Pedaudiology, University Hospital and Medical Faculty, RWTH Aachen University, Pauwelsstraße 30, Aachen, 52074, Germany, 49 2418038717; 2 Phoniatrics and Pedaudiology, Medical Faculty, RWTH Aachen University, Aachen, Germany

**Keywords:** medical students, e-learning, digital learning, e-learning tool, augmentative and alternative communication, AAC, teaching, phoniatrics

## Abstract

We developed an e-learning tool in augmentative and alternative communication (AAC) for medical students in Germany and tested it at Rhenish-Westphalian Technical University, Aachen. Our cross-sectional evaluation study is an innovative approach since the topic is yet to be implemented in medical education. Other AAC tools do not target medical students. Our study underlines the importance of AAC for medical doctors. Universities should include AAC in their lectures to prepare medical students for their clinical practice. The “*AAC*HEN” tool is a first step.

## Introduction

Augmentative and alternative communication (AAC) is the study of communicative strategies for patients who cannot speak [1], including the use of body language, symbol-boards, or technological devices [2,3]. Patients who use AAC are most common in medical fields related to speech and language such as phoniatrics, pediatrics, neurology, and otorhinolaryngology. However, every medical doctor may treat patients with complex communication needs. Therefore, knowledge in AAC is relevant in all medical specialties. Hurtig et al [4] show a lack of training in the field when implementing AAC in intensive care. What tools are available to meet the need for instruction? In a previous study, we gained insight into existing AAC tools for medical students and professionals [5]. No free-access, audio-visual, German online course with a knowledge quiz was found. Now, we fill this gap with the “*AAC*HEN” tool. The objectives of this study are to (1) underline the importance of AAC for medical doctors, (2) assess the students’ evaluations of our tool, and (3) give recommendations for future application in medical education.

## Methods

### Ethical Considerations

This study involved human participants and was reviewed and approved by the ethics committee of the Medical Faculty at Rheinisch-Westfälische Technische Hochschule (RWTH; Rhenish-Westphalian Technical University in English) Aachen University Hospital (no EK 24‐022; registration no CTC-A 24‐035). The students were informed about the methods and objectives of this study through a brief presentation. An information sheet was available in paper form and as a digital document. Following the provision of information, participants signed a consent form about the collection and analysis of the data. A full data protection impact assessment was not required since the feedback form was anonymous. Participation was voluntary, not compensated, and could be ended at any time. Identification of individual participants is not possible in any parts of this manuscript or supplementary material.

### Study Design: Setting, Participants, Recruitment, and Sample Size

**Figure 1.**
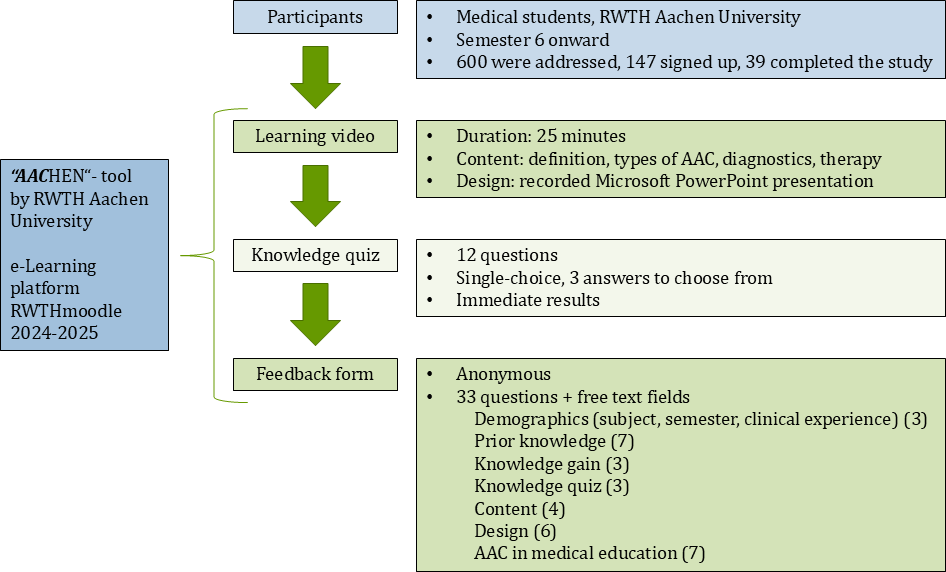
Overview of this study. The numbers in parentheses represent the number of questions. AAC: augmentative and alternative communication; RWTH: Rheinisch-Westfälische Technische Hochschule.

The tool was developed at the University Hospital and Medical Faculty RWTH Aachen from October 2023 until May 2024. Recruitment and evaluation lasted from June 2024 until August 2025. The study design was cross-sectional as students gave feedback after having worked through the tool. The inclusion criterion was the enrollment in the medical study course from semester 6 onward. We used convenience sampling for recruiting (ie, announcing in lectures). Approximately 600 students were addressed, 147 signed up, and 39 completed the study (Figure 1 and Multimedia Appendix 1).

### The “*AAC*HEN” Tool: Development, Review, Duration, Content, and Design

The tool consists of a learning video and a knowledge quiz (Multimedia Appendix 2). It was developed by author JB after she studied the literature and reviewed by medical doctors and speech-language pathologists. The video is a presentation of 25 minutes. The knowledge quiz is announced at the beginning. The presentation covers types of AAC, patients who could benefit from AAC, and which patient could benefit from what type of AAC (Multimedia Appendix 3).

### Data Assessment, Measurement, and Statistical Analysis

The students’ evaluations were assessed with a feedback form on RWTHmoodle (Multimedia Appendix 4). Levels of agreement to statements pertaining to the importance for medical doctors, inclusion in the curriculum, knowledge gain, and benefit were measured on Likert scales. Content, design, and prior knowledge were measured using the German grading system. Effects of the knowledge quiz were assessed using a multiple-choice format. Knowledge quiz results and feedback form data were analyzed. Means and confidence intervals were calculated using R software (version 4.5.2; R Foundation for Statistical Computing) (Multimedia Appendix 1).

## Results

Almost all students (38/39, 97.4%; 95% CI 86.5%‐99.9%) agreed to AAC being an important topic for medical doctors and the majority agreed that it should be included in their curriculum (30/39, 76.9%; 95% CI 60.7%‐88.9%). Almost none of the students had ever heard of AAC or had an idea of it (38/39, 97.4%; 95% CI 86.5%‐99.9%). Almost all gained knowledge from our tool (38/39, 97.4%; 95% CI 86.5%‐99.9%) and perceived this knowledge to be beneficial for their future clinical practice (37/39, 94.9%; 95% CI 82.7%‐99.4%). The announcement of the knowledge quiz had positive effects; students paid more attention (27/39, 69.2%; 95% CI 52.4%‐83%) and memorized the content better (21/39, 53.9%; 95% CI 37.2%‐69.9%). Most of the students stated that the “*AAC*HEN” tool should be a fixed part in their curriculum (29/39, 74.4%; 95% CI 57.9%‐87%). The “*AAC*HEN” tool received “very good” grades in terms of content (mean value 1.3, 95% CI 1.1‐1.6) and design (mean value 1.2, 95% CI 1‐1.3). The mean value of correct results in the knowledge quiz was 92.3% (95% CI 90.2%‐94.3%) (Table 1, Table 2, and Multimedia Appendix 5).

**Table 1.** Students’ answers in the feedback form (N=39).

	Agreement		Neutral, n (%)	Disagreement		Proportion agreement, n (%)	95 % CI (%)
	Yes, n (%)	Rather yes, n (%)		Rather no, n (%)	No, n (%)		
Importance for MD^a^	26 (66.7)	12 (30.8)	1 (2.6)	0 (0)	0 (0)	38 (97.4)	86.5‐99.9
AAC^b^ in curriculum	14 (35.9)	16 (41.0)	9 (23.1)	0 (0)	0 (0)	30 (76.9)	60.7‐88.9
Knowledge gain “AACHEN”	34 (87.2)	4 (10.3)	1 (2.6)	0 (0)	0 (0)	38 (97.4)	86.5‐99.9
Knowledge gain benefit MD	21 (53.9)	16 (41.0)	2 (5.1)	0 (0)	0 (0)	37 (94.9)	82.7‐99.4
“AACHEN” in curriculum	14 (35.9)	15 (38.5)	8 (20.5)	2 (5.1)	0 (0)	29 (74.4)	57.9‐87

a MD: medical doctors.

b AAC: augmentative and alternative communication.

**Table 2.** Students’ answers in the feedback form (N=39).

	1=Very good, n (%)	2=Good, n (%)	3=Satisfactory, n (%)	4=Sufficient, n (%)	5=Poor, n (%)	6=Deficient, n (%)	Value of grades, 1-6, mean (95% CI)
Prior knowledge in AAC	1 (2.6)	4 (10.3)	6 (15.4)	14 (35.9)	9 (23.1)	5 (12.8)	4.1 (3.6-4.5)
Content of “AACHEN”	30 (76.9)	8 (20.5)	0 (0)	0 (0)	0 (0)	1 (2.6)	1.3 (1.1-1.6)
Design of “AACHEN”	33 (84.6)	6 (15.4)	0 (0)	0 (0)	0 (0)	0 (0)	1.2 (1-1.3)

a AAC: augmentative and alternative communication.

## Discussion

### Principal Findings

We are not surprised that most of the students were not familiar with AAC since the topic is yet to be implemented in medical education. It is gratifying to note that the feedback about our tool was very positive and that the students gained knowledge from it. Although our study had a small sample size, it underlines the importance of AAC for medical doctors. While our research focuses on the testing of an e-learning tool, other studies address the importance of including the voices of AAC users in the instructional process [6,7]. We support a previously stated interest of learners in first-hand experience with AAC users [8] (Multimedia Appendix 6).

### Future Directions

We plan to combine our “*AAC*HEN” tool with an in-person lecture, where different types of AAC can be explored in cooperation with AAC users. The “*AAC*HEN” tool will be added to the RWTH Aachen toolbox app [9]. In addition, it could be added to an AAC online platform for German-speaking learners [10]. We would like to provide nationwide access to our tool and collaborate with other medical faculties.

### Conclusion

Our study underlines the importance of AAC in medicine and the lack of students’ knowledge in the field. AAC is not yet taught to medical students, and appropriate e-learning tools do not yet exist. Our “*AAC*HEN” tool fills a significant gap in the teaching of AAC. It can be included in lectures and be combined with practical instructions to improve medical education.

## Supplementary material

10.2196/88173 Multimedia Appendix 1 Detailed description of methods.

10.2196/88173 Multimedia Appendix 2 Knowledge quiz (original German version).

10.2196/88173 Multimedia Appendix 3 Detailed description of the “*AAC*HEN” tool.

10.2196/88173 Multimedia Appendix 4 Feedback form (original German version).

10.2196/88173 Multimedia Appendix 5 Detailed results.

10.2196/88173 Multimedia Appendix 6 Detailed discussion.

## Abbreviations

AAC: augmentative and alternative communication
RWTH: Rheinisch-Westfälische Technische Hochschule
