# Supplementary material for: “AACHEN” e-Learning Tool in Augmentative and Alternative Communication for Medical Students in Germany: Cross-Sectional Evaluation Study
Source: JMIR Med Educ. 2026 Apr 29;12:e88173. doi: 10.2196/88173 (PMC13127592; doi:10.2196/88173)
Supplement: Multimedia Appendix 1 [file mededu-v12-e88173-s001.docx]

**Recruitment, Procedure, and Sample Size**

Medical students from RWTH Aachen University were recruited to participate in our study from June 2024 until August 2025. At the end of a phoniatrics-lecture in semester 6, we informed the students about the opportunity to participate in our e-learning study. Students who were interested were provided with additional information about the study and instructions on how to sign up. This procedure was carried out with two courses in total, in year 2024 and in year 2025. In addition, we asked other medical students that met the inclusion criteria to participate in our study. Furthermore, some students recommended our study to their peers who were not present at the lecture. All actions considered, approximately 600 medical students were addressed. 147 of them were interested in participating and sent us their signed consent form. They were added to the “***AAC***HEN”-tool on our e-learning platform RWTHmoodle, having access to the learning video, the knowledge quiz and the feedback form. Thus, they could participate from anywhere and anytime with their personal login on their personal devices or using a university-owned computer. The tool was available from June 2024 to August 2025. The study design was cross-sectional as students gave feedback once after having worked through the tool. Approximately six weeks after each student’s signup, a reminder email was sent to encourage students to participate. 147 medical students had access to “***AAC***HEN” and 39 of them completed the study. The study could be terminated at any time. Only complete feedback forms were added to the dataset. Participation was not compensated and voluntary. The study was estimated to take 40 minutes to complete.

**Inclusion and Exclusion Criteria for Participants**

Participants must be at least 18 years old and study medicine at RWTH Aachen University from semester 6 onwards. We excluded semester 1-5 because we wanted the students to have a basic understanding of their field.

**Feedback Form**

After watching the learning video and performing the knowledge quiz, students were asked to give anonymous feedback. The completion of the feedback form took approximately 10 minutes. The feedback form was accessible to the students as soon as they checked a box to confirm that they had watched the video. The feedback form consisted of 33 questions and free text fields. First, 3 obligatory demographic questions were asked about the students’ subject, semester, and years of experience. The students’ subject was assessed because the “***AAC***HEN”-tool was also available for students of speech-language pathology. Since it is common for speech-language pathology students to have clinical experience prior to the Master study course, “years of experience” were asked. All other answers were optional. The questions covered prior knowledge, knowledge gain from the learning video, the learning video’s content and design, and AAC in medical education. We used different levels of measurement:

*3- or 5-point Likert scales (single choice):* The level of agreement for certain statements was rated on a 5-point scale from “yes,” “rather yes,” “neutral,” “rather no” to “no.” We chose this scale because we wanted a clear “neutral” option, thus eliminating the need for students to make a binary decision between "yes" and "no." To state, whether students have heard of AAC before, they could choose from a 3-point scale from “rather well,” “an idea of” to “never heard.”

*German grading system (single choice):* The scale displays grades from very good (1), good (2), satisfactory (3), sufficient (4), poor (5) to deficient (6). We chose this scale because it is familiar to students in Germany.

*Multiple-choice options:* Some questions could be answered by choosing multiple options (eg, what effects the knowledge quiz had on the students). Here, a “none of the above” or “I do not know” option was included to ensure the students did not choose an option they did not fully support.

*Free text fields*: To explain certain answers or to provide improvement suggestions, students could type into free text fields.

**Statistical Analysis**

The data of the feedback form were analyzed. Means and confidence intervals were calculated using R software (version 4.5.2; R Foundation for Statistical Computing).

*3- or 5-point Likert scales (single choice):* Confidence intervals were calculated for a proportion (eg, agreement) by dichotomizing the answers.

*German grading system (single choice):* Confidence intervals were calculated of mean values by treating the scale quasi-metric.

*Multiple-choice options*: Confidence intervals were calculated for the proportion of students having selected each category.
