# Supplementary material for: “AACHEN” e-Learning Tool in Augmentative and Alternative Communication for Medical Students in Germany: Cross-Sectional Evaluation Study
Source: JMIR Med Educ. 2026 Apr 29;12:e88173. doi: 10.2196/88173 (PMC13127592; doi:10.2196/88173)
Supplement: Multimedia Appendix 2 [file mededu-v12-e88173-s002.docx]

**Wissensabfrage**

Es folgen Fragen mit Antwortmöglichkeiten. Nur eine Antwort ist korrekt.

- Pausieren Sie das Video, nachdem eine Frage gestellt wurde.
- Lesen Sie sich die Fragen durch und überlegen, was die richtige Antwort sein könnte.
- Setzen Sie dann das Video fort und sehen, ob Sie richtig liegen. Die korrekten Antworten werden markiert.
- Zählen Sie Ihre richtigen Antworten.

**Grundsätzliches**

FRAGE 1

Welche Aussage ist korrekt?

- Der Einsatz von UK sollte gut überlegt sein, denn er kann die Lautsprache hemmen.
- UK sollte erst dann eingesetzt werden, wenn alle anderen Therapien erfolglos waren.
- Atmen ist die einzige Voraussetzung für UK.

FRAGE 2

Welche Aussage ist korrekt?

- Nur eine Form der UK soll eingeübt werden, da sonst zu viele Wahrnehmungskanäle gleichzeitig stimuliert werden.
- In der UK gilt das Prinzip der Multimodalität, nach dem mehrere Kommunikationskanäle gleichzeitig trainiert werden sollen.
- Jede/r UK-Patient*in muss Gebärdensprache lernen, damit er sich auch im Ausland verständlich machen kann.

FRAGE 3

Welche Aussage ist korrekt?

- UK gibt es bislang nur in Deutschland, wird aktuell allerdings immer mehr weltweit etabliert.
- UK spielt eine zentrale Rolle für die Teilhabe.
- Mit Ansteuerung ist immer Augensteuerung gemeint.

**Formen der UK**

FRAGE 4

Was zählt zu den körpereigenen Kommunikationsformen?

- Blickbewegungen
- Medizinischer Notfallpass
- Individualisierter Vorlesestift

FRAGE 5

Ein iPad mit Talker App ist…

- …eine dynamische Kommunikationshilfe, da durch Betätigen eines Feldes eine neue Oberfläche erscheint.
- …ein „low-tech“ Gerät, da es als Einstieg in die technischen Kommunikationshilfen gilt.
- …eine statische Kommunikationshilfe, da der/die Patient*in das iPad immer am Körper tragen sollte.

FRAGE 6

Welches der folgenden Hilfsmittel sind statische Kommunikationshilfen?

- Ich-Bücher, die wetterfest am Rollstuhl befestigt sind
- Tastaturen mit Display und Wortvervollständigung
- Sprechende Tasten oder „low-tech“ Talker, mit denen vorgefertigte Aussagen abgespielt werden können

**UK Patient*innen**

FRAGE 7

Welche Aussage ist korrekt?

- Patient*innen mit Aphasie können von UK profitieren, auch wenn sie noch schreiben können.
- Bei Patient*innen mit Amyotropher Lateralsklerose wird mit UK begonnen, sobald die Lautsprache nicht mehr verständlich ist.
- Bei einem global entwicklungsverzögerten dreijährigen Jungen mit ausbleibender Lautsprache sollte mit UK noch gewartet werden.

FRAGE 8

Welche Aussage ist korrekt?

- Bei Patient*innen mit Autismus-Spektrum-Störung ist UK ungeeignet, da die Interaktion gestört ist.
- Bei Trisomie 21 wird UK häufig eingesetzt, obwohl Lautsprache vorhanden ist.
- Auf Intensivstationen wird UK nicht angewandt, da zunächst geschaut werden muss, ob sich der Allgemeinzustand der Patient*innen bessert.

FRAGE 9

Welche Aussage ist korrekt?

- Patient*innen, die eine Kommunikationshilfe haben, behalten diese ein Leben lang.
- Kommunikationshilfen sollten regelmäßig angepasst werden.
- Patient*innen, bei denen die Kommunikationshilfe vom Kostenträger abgelehnt wurde, benötigen auch keine.

**Patient‘*innen einer UK-Form zuordnen**

FRAGE 10

Ein dreijähriges Kind mit Infantiler Zerebralparese und Hemiparese kann nicht laufen und sprechen. Es hat noch kein Konzept von Selbstwirksamkeit. Welche der folgenden Hilfsmittel kommt in Frage?

- Talker mit Kopfsteuerung
- Geräte zur Ursache-Wirkungsanbahnung
- Gebärdensprache

FRAGE 11

Ein 55-jähriger Mann mit Amyotropher Lateralsklerose verliert zunehmend die Fähigkeit, sich lautsprachlich zu verständigen. Seine Motorik lässt ebenso zunehmend nach. Sein Denkvermögen ist nicht beeinträchtigt. Welches der folgenden Hilfsmittel kommt in Frage?

- Talker mit Augensteuerung
- Gebärdensprache
- Mikrofon- und Lautsprecheranlage

FRAGE 12

Eine im Vokaltrakt frischoperierte, 30-jährige Frau hat eine taube, geschwollene Zunge, Missempfindungen im Gesichtsbereich und spricht unverständlich. Prognostisch wird die Schwellung in wenigen Tagen vollständig abgeklungen sein. Welche Hilfsmittel bieten Sie an?

- Keine, da die Patientin in wenigen Tagen wieder sprechen kann
- Alexa und iPod mit Spotify
- Blatt und Stift / schriftbasiertes Gerät und ggf. Notfallpass
