# Supplementary material for: “AACHEN” e-Learning Tool in Augmentative and Alternative Communication for Medical Students in Germany: Cross-Sectional Evaluation Study
Source: JMIR Med Educ. 2026 Apr 29;12:e88173. doi: 10.2196/88173 (PMC13127592; doi:10.2196/88173)
Supplement: Multimedia Appendix 3 [file mededu-v12-e88173-s003.docx]

**The Aachen AAC e-Learning Tool “*AAC*HEN”**

We created an AAC e-learning tool and named it “***AAC***HEN” as a merged term of “AAC” and “Aachen”, its faculty-city of origin. It consists of a learning video and a knowledge quiz.

**Development**

The tool was developed by author JB who specializes in the field of AAC. The process lasted approximately from October 2023 until May 2024. Literature review, experience in teaching and clinical practice in AAC, and insights of a previous study about the structures of existing tools were the basis of the tools’ development.

**Review**

To ensure the best content and quality possible, the tool was reviewed by our team at the *Clinic for Phoniatrics, Pedaudiology & Communication Disorders*, consisting of medical doctors and speech-language pathologists. Since August 2025, the clinic’s name has changed to: *Clinic for Otorhinolaryngology, Phoniatrics & Pedaudiology*.

**Learning Video**

*Design*

In terms of design, the learning video was a recorded Microsoft PowerPoint presentation with the lecturer’s original voice-over and visual appearance. The presentation slides were prepared for the specific purpose of this study. They contained texts, pictures (photos and drawings), and diagrams. Author JB created the presentation and performed as the speaker in the video. The video was recorded, cut, and finished in a studio at the *Audio-Visual-Media-Center* of RWTH Aachen University. The video’s duration is approximately 25 minutes.

*Content*

With respect to content, the learning outcomes were based on the knowledge that medical doctors need to treat patients with complex communication needs. We defined three learning outcomes: after having watched the video, students should know (1) the types of AAC, (2) which patients may benefit from AAC, and (3) what type of AAC fits to which kind of patient. The video starts with the announcement that the students can test their learning progress in a knowledge quiz at the end of the video. A definition of AAC is given and the types of AAC are explained. Furthermore, patients are identified that could benefit from AAC, mentioning symptoms and common diagnoses. In addition, examples are given for which patient could benefit from what type of AAC. References are given for further reading.

**Knowledge Quiz**

The knowledge quiz was announced at the beginning of the video. At the end of the video, students were instructed as follows: *“Questions with possible answers follow. Only one answer is correct. Pause the video after each question. Read the question and choose an answer. Continue the video to see if your answer is correct. Count your correct answers.”* A total of 12 questions were asked and the correct answers were provided immediately. Of these 12 questions, 3 pertained to “general knowledge in AAC,” 3 to “types of AAC,” 3 to “AAC patients,” and 3 to “patients and their types of AAC.” The guessing probability was 33% in a 1 out of 3-choice. All questions were specifically invented for the purpose of this study and were addressed in the learning video. To check for correctness and plausibility, we asked our colleagues to review our questions. The duration of the knowledge quiz depended on the participant’s pace. We assume it took approximately 10 minutes.

To get an understanding of the knowledge quiz, we would like to give three examples of the questions:

(1) *A 30-year-old female who has recently undergone surgery on her vocal tract has a numb, swollen tongue, paresthesia in her face, and shows unintelligible speech. The swelling is expected to subside completely in a few days. What communication aids do you offer? A)* *None, as the patient will be able to speak again in a few days, B) Alexa and iPod with Spotify, C) Paper and pencil or a text-based device and a sheet with emergency information if needed* (correct answer)*;*

(2) *A 55-year-old male with amyotrophic lateral sclerosis is progressively losing the ability to speak. His motoric skills are also declining. Cognition is not affected. Which of the following communication aids would fit? A) Speech generating device with eye gaze* (correct answer)*, B) Sign language, C) Microphone and speaker;*

(3) *Which statement is correct? A) AAC is not an option for patients with autism spectrum disorder because their interaction is impaired, B) AAC is often used in patients with Trisomy 21, even though they are able to speak* (correct answer)*, C) AAC is not used in intensive care because the patient’s general state of health needs to improve first.*
