# Supplementary material for: “AACHEN” e-Learning Tool in Augmentative and Alternative Communication for Medical Students in Germany: Cross-Sectional Evaluation Study
Source: JMIR Med Educ. 2026 Apr 29;12:e88173. doi: 10.2196/88173 (PMC13127592; doi:10.2196/88173)
Supplement: Multimedia Appendix 4 [file mededu-v12-e88173-s004.pdf]

Evaluationsbogen

Weiter

Modus: Anonym

Zunächst benötigen wir Angaben zu Ihrem Studiengang, Ihrem Fachsemester und Ihrer Berufserfahrung:

Was studieren Sie?

- ○ Humanmedizin
- ○ Lehr- und Forschungslogopädie

In welchem Semester sind Sie? (1 - 30)

Wie viel Berufserfahrung haben Sie?

- ○ Keine
- ○ Bis zu einem Jahr
- ○ Ein bis drei Jahre
- ○ Mehr als drei Jahre

Uns interessiert Ihr Vorwissen im Bereich UK:

Hatten Sie vor diesem Lernvideo schon einmal von UK gehört?

- ○ Nein, noch nie
- ○ Ja, ich wusste grob, was UK ist
- ○ Ja, ich wusste schon ziemlich ziemlich genau, was UK ist

Konnten Sie vor diesem Lernvideo definieren, was UK ist?

- ◉ Nicht gewählt
- ○ (1)Nein
- ○ (2)Eher nein
- ○ (3)Teils-teils
- ○ (4)Eher ja
- ○ (5)Ja

Konnten Sie vor diesem Lernvideo unterschiedliche Formen der UK benennen?

- ◉ Nicht gewählt
- ○ (1)Nein
- ○ (2)Eher nein
- ○ (3)Teils-teils
- ○ (4)Eher ja
- ○ (5)Ja

Wussten Sie vor diesem Lernvideo, welche Patient*innen von UK profitieren können?

- ◉ Nicht gewählt
- ○ (1)Nein
- ○ (2)Eher nein
- ○ (3)Teils-teils
- ○ (4)Eher ja
- ○ (5)Ja

Wussten Sie vor diesem Lernvideo, welche Patient*innen von welcher Kommunikationshilfe profitieren können?

- ◉ Nicht gewählt
- ○ (1)Nein
- ○ (2)Eher nein
- ○ (3)Teils-teils
- ○ (4)Eher ja

○ (5)Ja

Wie gut war Ihr Vorwissen bezüglich UK insgesamt? Bitte geben Sie sich eine Schulnote.

◉ Nicht gewählt
○ (1)Sehr gut
○ (2)Gut
○ (3)Befriedigend
○ (4)Ausreichend
○ (5)Mangelhaft
○ (6)Ungenügend

Wenn Sie bereits Vorwissen bezüglich UK hatten, woher hatten Sie es? (Es können mehrere Antworten ausgewählt werden.)

☐ Lehrveranstaltung im Studium
☐ Unterricht in der Ausbildung
☐ Berufliche Tätigkeiten
☐ Praktikum
☐ Freunde / Angehörige / Bekannte, die UK-Nutzer*innen sind
☐ Sonstiges

Bitte erläutern Sie, woher Sie Ihr Vorwissen haben, z. B. in welchem Bereich das Praktikum war oder im Rahmen welcher Lehrveranstaltung Sie bereits von UK gehört haben.

Bei den nun folgenden Fragen geht es um Ihren Informationsgewinn durch dieses Lernvideo:

Wie viele Fragen der Wissensabfrage konnten Sie richtig beantworten? (0 - 12)

Hat dieses Lernvideo Ihr Wissen bezüglich UK erweitert?

◉ Nicht gewählt
○ (1)Nein
○ (2)Eher nein
○ (3)Teils-teils
○ (4)Eher ja
○ (5)Ja

Falls Sie durch dieses Lernvideo Ihr Wissen im Bereich UK erweitern konnten, ist dieses Wissen für Sie ein fachlicher Zugewinn als angehende Ärztin / angehender Arzt / Logopädin / Logopäde?

◉ Nicht gewählt
○ (1)Nein
○ (2)Eher nein
○ (3)Teils-teils
○ (4)Eher ja
○ (5)Ja

Bitte bewerten Sie nun die Inhalte dieses Lernvideos:

Die unterschiedlichen Formen der UK wurden verständlich dargestellt.

◉ Nicht gewählt
○ (1)Nein
○ (2)Eher nein
○ (3)Teils-teils
○ (4)Eher ja
○ (5)Ja

Es wurde deutlich, welche Patient*innen von UK profitieren können.

- ◉ Nicht gewählt
- ○ (1)Nein
- ○ (2)Eher nein
- ○ (3)Teils-teils
- ○ (4)Eher ja
- ○ (5)Ja

Es wurde deutlich, welche Kommunikationshilfe für welche Patient*innen in Frage kommen könnte.

- ◉ Nicht gewählt
- ○ (1)Nein
- ○ (2)Eher nein
- ○ (3)Teils-teils
- ○ (4)Eher ja
- ○ (5)Ja

Welche Schulnote geben Sie für den Inhalt dieses Lernvideos?

- ◉ Nicht gewählt
- ○ (1)Sehr gut
- ○ (2)Gut
- ○ (3)Befriedigend
- ○ (4)Ausreichend
- ○ (5)Mangelhaft
- ○ (6)Ungenügend

Haben Sie Verbesserungsvorschläge für den Inhalt dieses Lernvideos?

Die nun folgenden Fragen beziehen sich auf die Wissensabfrage.

Hatte die Ankündigung der Wissensabfrage Einfluss auf die Intensität Ihres Lernens? Bitte markieren Sie, welche Aussage(n) zutrifft / zutreffen.

- ☐ Die angekündigte Wissensabfrage hat mich dazu motiviert, mir die Inhalte zu merken.
- ☐ Die angekündigte Wissensabfrage hat mich dazu motiviert, das Video vollständig anzusehen.
- ☐ Durch die angekündigte Wissensabfrage habe ich das Video aufmerksam verfolgt.
- ☐ Die Ankündigung der Wissensabfrage hat nichts in mir ausgelöst.
- ☐ Ich weiß es nicht.
- ☐ Sonstiges

Hier können Sie Ihre vorherige Antwort erläutern.

Welche Aussage(n) trifft / treffen bzgl. der Wissensabfrage Ihrer Meinung nach zu?

- ☐ Durch die Beantwortung der Fragen wurden mir manche Zusammenhänge klarer.
- ☐ Ich fand gut, dass ich mich aktiv mit den Lerninhalten auseinandersetzen konnte.
- ☐ Ich fand gut, dass ich mein Wissen / meinen Lernfortschritt direkt testen konnte.
- ☐ Insgesamt fand ich die Wissensabfrage hilfreich.
- ☐ Mir hätte es gereicht, das Lernvideo ohne die Wissensabfrage anzusehen.
- ☐ Die Wissensabfrage war Zeitverschwendung.
- ☐ Sonstiges

Hier können Sie Ihre vorherige Antwort erläutern.

Wie beurteilen Sie den Schweregrad der Fragen?

- ◉ Nicht gewählt
- ○ (1)Leicht
- ○ (2)Eher leicht
- ○ (3)Genau richtig
- ○ (4)Eher schwer
- ○ (5)Schwer

Haben Sie Anregungen oder Kommentare zu der Wissensabfrage?

Bitte bewerten Sie nun die Gestaltung dieses Lernvideos:

Die Fotos waren passend ausgewählt, so dass ich mir gut vorstellen konnte, um was es geht.

- ◉ Nicht gewählt
- ○ (1)Nein
- ○ (2)Eher nein
- ○ (3)Teils-teils
- ○ (4)Eher ja
- ○ (5)Ja

Die Texte waren verständlich und auf den Punkt gebracht.

- ◉ Nicht gewählt
- ○ (1)Nein
- ○ (2)Eher nein
- ○ (3)Teils-teils
- ○ (4)Eher ja
- ○ (5)Ja

Ich konnte der Sprecherin gut folgen. Die Aussagen waren klar und verständlich.

- ◉ Nicht gewählt
- ○ (1)Nein
- ○ (2)Eher nein
- ○ (3)Teils-teils
- ○ (4)Eher ja
- ○ (5)Ja

Das Foliendesign war ansprechend.

- ◉ Nicht gewählt
- ○ (1)Nein
- ○ (2)Eher nein
- ○ (3)Teils-teils
- ○ (4)Eher ja
- ○ (5)Ja

Die Qualität dieses Lernvideos war gut (Auflösung, Audio).

- ◉ Nicht gewählt
- ○ (1)Nein
- ○ (2)Eher nein

◯ (3)Teils-teils
◯ (4)Eher ja
◯ (5)Ja

Welche Schulnote geben Sie für die Gestaltung dieses Lernvideos?

◉ Nicht gewählt
◯ (1)Sehr gut
◯ (2)Gut
◯ (3)Befriedigend
◯ (4)Ausreichend
◯ (5)Mangelhaft
◯ (6)Ungenügend

Haben Sie Verbesserungsvorschläge zur Gestaltung dieses Lernvideos?

Es folgen Fragen zum Thema UK in der Lehre.

Finden Sie das Thema UK wichtig für Logopäd*innen?

◉ Nicht gewählt
◯ (1)Nein
◯ (2)Eher nein
◯ (3)Teils-teils
◯ (4)Eher ja
◯ (5)Ja

Sie können hier gerne Ihre vorherige Antwort erläutern.

Finden Sie das Thema UK wichtig für Ärzt*innen?

◉ Nicht gewählt
◯ (1)Nein
◯ (2)Eher nein
◯ (3)Teils-teils
◯ (4)Eher ja
◯ (5)Ja

Sie können hier gerne Ihre vorherige Antwort erläutern.

Sollte das Thema UK ein fester Bestandteil in der Lehre Ihres Studiengangs (Masterstudiengang Lehr- und Forschungslogopädie bzw. Modellstudiengang Humanmedizin) werden?

◉ Nicht gewählt
◯ (1)Nein
◯ (2)Eher nein
◯ (3)Teils-teils
◯ (4)Eher ja

○ (5)Ja

Sie können hier gerne Ihre vorherige Antwort erläutern.

Wenn UK ein Bestandteil Ihres Studiengangs sein soll, wie sollte UK gelehrt werden? (Es können mehrere Antworten ausgewählt werden.)
☐ Vorlesung in Präsenz
☐ Onlineveranstaltungen (z. B. über Zoom)
☐ Lernvideos (z. B. in Moodle)
☐ Praktikum
☐ Übung
☐ Sonstiges

Sie können hier gerne Ihre vorherige Antwort erläutern.

Sollte dieses Lernvideo (oder eine verbesserte Version dessen) ein fester Bestandteil in der Lehre Ihres Studiengangs (Masterstudiengang Lehr- und Forschungslogopädie bzw. Modellstudiengang Humanmedizin) werden?
◉ Nicht gewählt
○ (1)Nein
○ (2)Eher nein
○ (3)Teils-teils
○ (4)Eher ja
○ (5)Ja

Es gibt Überlegungen, eine fächerübergreifende Lern-App zu entwickeln, in der allerlei "Tools" gesammelt werden für Studierende und Berufstätige in den Bereichen Logopädie, HNO und Phoniatrie.

Sollte dieses Lernvideo (oder eine verbesserte Version dessen) einer solchen App hinzugefügt werden?
◉ Nicht gewählt
○ (1)Nein
○ (2)Eher nein
○ (3)Teils-teils
○ (4)Eher ja
○ (5)Ja

Hier können Sie Ihre vorherige Antwort erläutern.

Welche Kanäle würden Sie nutzen, um sich Wissen im Bereich UK anzueignen? (Es können mehrere Antworten ausgewählt werden.)
☐ Keine, ich würde mich nicht damit beschäftigen.
☐ Ich würde Webseiten zu dem Thema durchstöbern.
☐ Ich würde Podcasts zu dem Thema anhören.
☐ Ich würde Apps zu dem Thema herunterladen.
☐ Ich würde Video-Plattformen (z. B. YouTube) durchsuchen.
☐ Ich würde soziale Netzwerke (z. B. Instagram, Facebook) zu dem Thema durchsuchen.
☐ Ich würde frei zugängliche, kostenlose Online-Kursen anschauen / machen.
☐ Ich würde kostenpflichtige Online-Kurse anschauen / machen.

☐ Ich würde Fortbildungen in Präsenz besuchen.
☐ Ich würde auf Tagungen fahren (z. B. von der Gesellschaft für UK).
☐ Ich würde Bücher zu dem Thema lesen.
☐ Ich würde wissenschaftliche Artikel zu dem Thema lesen.

Fallen Ihnen noch weitere Kanäle ein, die Sie nutzen würden, um an Informationen zum Thema UK zu kommen?

Zum Abschluss noch eine allgemeine Frage:

Haben Sie Vorschläge, Ideen, Kommentare zu diesem Evaluationsbogen oder unserer Studie? Bitte teilen Sie uns gerne Ihre Gedanken mit!

notwendig

Weiter
