# Supplementary material for: “AACHEN” e-Learning Tool in Augmentative and Alternative Communication for Medical Students in Germany: Cross-Sectional Evaluation Study
Source: JMIR Med Educ. 2026 Apr 29;12:e88173. doi: 10.2196/88173 (PMC13127592; doi:10.2196/88173)
Supplement: Multimedia Appendix 5 [file mededu-v12-e88173-s005.docx]

**Demographics (Table 1)**

39 medical students participated in our study. Most of them were in semester 6 (22/39, 56.4%) or 7 (15/39, 38.5%) and had no clinical experience (24/39, 61.5%). 4 (10.3%) had up to one year of clinical experience, 6 (15.4%) between one and three years, and 5 (12.8%) more than three years.

| What is your study course? | n (%) |
| --- | --- |
| Medicine | 39 (100) |
| Speech-language pathology | 0 (0) |
|  | |
| What is your semester? | n (%) |
| 6 | 22 (56.4) |
| 7 | 15 (38.5) |
| 8 | 2 (5.1) |
|  | |
| How much clinical experience do you have? | n (%) |
| None | 24 (61.5) |
| Up to 1 year | 4 (10.3) |
| 1 to 3 years | 6 (15.4) |
| More than 3 years | 5 (12.8) |

Table 1: Demographics.

**Prior Knowledge (Table 2)**

Of the 39 participants, 15 (38.5%) had never heard of AAC before. 23 (59%) had an idea of what AAC is. Only 1 student (2.6%) knew rather well what AAC is. When asked how the students would rate their prior knowledge according to the German grading system, 1 (2.6%) answered “very good,” 4 (10.3%) “good,” 6 (15.4%) “satisfactory,” 14 (35.9%) “sufficient,” 9 (23.1%) “poor,” and 5 (12.8%) “deficient.”

Sources of prior knowledge were explained in the free text field as follows: AAC-using friends or relatives; clinical placements, internships, occupations in health care, or voluntary social year in neurology, pediatrics, intensive care, nursing, speech-language pathology, occupational therapy, paramedics, or at a center for special needs; lectures at university or vocational school; social media; television and movies, and via famous AAC-using personalities.

| Have you heard of AAC prior to this study? | n (%) | Proportion of insufficient knowledge:  97.4% (38/39)  95% CI: 86.5%-99.9% |
| --- | --- | --- |
| I knew rather well what AAC is. | 1 (2.6) |  |
| I had an idea of what AAC is. (insufficient) | 23 (59) |  |
| I have never heard of AAC. (insufficient) | 15 (38.5) |  |
|  | | |
| How would you rate your prior knowledge in AAC? (German grading system 1-6) | n (%) | Mean value: 4.1  95% CI: 3.6-4.5 |
| Very good (1) | 1 (2.6) |  |
| Good (2) | 4 (10.3) |  |
| Satisfactory (3) | 6 (15.4) |  |
| Sufficient (4) | 14 (35.9) |  |
| Poor (5) | 9 (23.1) |  |
| Deficient (6) | 5 (12.8) |  |
|  | | |
| Please explain the sources of your prior knowledge in AAC  (free text field) | | |
| AAC-using friends or relatives; clinical placements, internships, occupations in health care, or voluntary social year in neurology, pediatrics, intensive care, nursing, speech-language pathology, occupational therapy, paramedics, or at a center for special needs; lectures at university or vocational school; social media; television and movies; via famous AAC using personalities | | |

Table 2: Prior knowledge; AAC = augmentative and alternative communication.

**Knowledge Gain (Table 3)**

Of the 39 students, 34 (87.2%) answered “yes” when asked if they gained knowledge from the learning video. Here, 4 (10.3%) chose “rather yes,” 1 (2.6%) “neutral,” and none “rather no” or “no.” When asked whether the gained knowledge would benefit them as a medical doctor candidate, 21 (53.9%) answered “yes,” 16 (41%) “rather yes,” and 2 (5.1%) “neutral.” Again, none chose “rather no” or “no.” When asked how many of the 12 questions of the knowledge quiz were answered correctly, 34 of the total 39 participants shared their results. 52.9% (18/34) had 11 questions correct , 26.5% (9/34) 12, and 20.6% (7/34) 10.

| Did you gain knowledge from our learning video? | n (%) | Proportion of agreement (“rather yes” or “yes”):  97.4% (38/39)  95% CI: 86.5%-99.9% |
| --- | --- | --- |
| No | 0 (0) |  |
| Rather no | 0 (0) |  |
| Neutral | 1 (2.6) |  |
| Rather yes | 4 (10.3) |  |
| Yes | 34 (87.2) |  |
|  | | |
| If you gained knowledge from our learning video, will it benefit you as a medical doctor candidate? | n (%) | Proportion of agreement (“rather yes” or “yes”):  94.9% (37/39)  95% CI: 82.7%-99.4% |
| No | 0 (0) |  |
| Rather no | 0 (0) |  |
| Neutral | 2 (5.1) |  |
| Rather yes | 16 (41) |  |
| Yes | 21 (53.9) |  |
|  | | |
| How many questions did you answer correctly in the knowledge quiz? | n (%) | Mean value of correct answers in %: 92.3%  95% CI: 90.2%-94.3% |
| 1-9 (<83% correct) | 0 (0) |  |
| 10 (83% correct) | 7 (20.6) |  |
| 11 (92% correct) | 18 (52.9) |  |
| 12 (100% correct) | 9 (26.5) |  |

Table 3: Knowledge gain; 34 knowledge quiz results

**Knowledge Quiz (Table 4)**

First, we wanted to know whether the announcement of the knowledge quiz affected the intensity of the students’ learning. Multiple answers were possible. 21 (53.9%) answered that the announced knowledge quiz motivated them to memorize the content. 21 (53.9%) answered that the announced knowledge quiz motivated them to watch the whole video. 27 (69.2%) said they paid attention to the video because of the announcement of the knowledge quiz. Only 2 (5.1%) said that the announcement did not affect them at all. None chose “I do not know.” In the free text fields, students wrote that due to the announced knowledge quiz, they felt more motivated, listened carefully, read the text thoroughly, and paused the video to take notes. It was also mentioned that the knowledge quiz resulted in a learning effect. 17 (43.6%) said that answering the questions made the video’s content clearer for them. 26 (66.7%) liked that they were actively thinking about the video’s content. 25 (64.1%) liked that they could test their learning progress immediately. 25 (64.1%) said that the knowledge quiz was helpful. Only 3 (7.7%) said that watching the video without performing the knowledge quiz would have been enough for them. The students explained that the knowledge quiz helped them memorize the facts and provided additional information. The questions were perceived as being easy. Students suggested increasing the questions’ difficulty gradually and offering an electronic quiz.

| Did the announcement of the knowledge quiz affect the intensity of your learning?  (multiple choice) | n (%) | 95% CI (%) |
| --- | --- | --- |
| The announced knowledge quiz motivated me to memorize the content. | 21 (53.9) | 37.2-69.9 |
| The announced knowledge quiz motivated me to watch the whole video. | 21 (53.9) | 37.2-69.9 |
| Because of the announced knowledge quiz, I paid attention to the video. | 27 (69.2) | 52.4-83 |
| The announced knowledge quiz did not affect me. | 2 (5.1) | 0.6-17.3 |
| I don’t know. | 0 (0) | 0-9 |
|  | | |
| If you wish to explain your previous answer, use this free text field | | |
| Motivation was higher; listened carefully and read the text thoroughly; important for learning effect; took notes and paused the video a couple of times | | |
|  | | |
| Which statement is applicable to you?  (multiple choice) | n (%) | 95% CI (%) |
| Answering the questions made things clearer for me. | 17 (43.6) | 27.8-60.4 |
| I liked that I was actively thinking about the video’s content. | 26 (66.7) | 49.8-80.9 |
| I liked that I could test my learning progress immediately. | 25 (64.1) | 47.2-78.8 |
| The knowledge quiz was helpful. | 25 (64.1) | 47.2-78.8 |
| The video without the knowledge quiz would have been enough for me. | 3 (7.7) | 1.6-20.9 |
|  | | |
| If you wish to explain your previous answer, use this free text field | | |
| Knowledge quiz helped memorize the facts; useful additional information and thoughts due to the knowledge quiz; questions were very easy; offering the knowledge quiz with a time delay to feel the need to memorize; being able to pause and go back to the video helped me process the content | | |
|  | | |
| What are your suggestions for improvement regarding the knowledge quiz?  (free text field) | | |
| Electronic quiz, interactive quiz, more difficult questions, a gradual increase in difficulty of the questions | | |

Table 4: Effects of knowledge quiz.

**Content (Table 5)**

When asked whether the types of AAC were presented in a comprehensible manner, 31 (79.5%) said “yes,” 8 (20.5%) “rather yes,” and none “no,” “rather no,” or “neutral.” 37 (94.9%) said the video made it clear which kinds of patients could benefit from AAC. Here, 2 (5.1%) said “rather yes” and none “no,” “rather no,” or “neutral.” 30 (76.9%) said the video clearly demonstrated what type of AAC may fit to which kind of patients. 9 (23.1%) said “rather yes” and none “no,” “rather no,” or “neutral.” When students were asked how they would rate the video’s content according to the German grading system, 30 (76.9%) gave the best grade “very good,” 8 (20.5%) the second-best grade “good,” and 1 (2.6%) the worst grade “deficient.” Students recommended including videos of patients using AAC and providing more technical details about speech generating devices.

| The types of AAC were presented in a comprehensible manner. | n (%) | Proportion of agreement  (“rather yes” or “yes”):  100% (39/39)  95% CI: 91%-100% |
| --- | --- | --- |
| No | 0 (0) |  |
| Rather no | 0 (0) |  |
| Neutral | 0 (0) |  |
| Rather yes | 8 (20.5) |  |
| Yes | 31 (79.5) |  |
|  | | |
| It was made clear which patients could benefit from AAC. | n (%) | Proportion of agreement (“rather yes” or “yes”):  100% (39/39)  95% CI: 91%-100% |
| No | 0 (0) |  |
| Rather no | 0 (0) |  |
| Neutral | 0 (0) |  |
| Rather yes | 2 (5.1) |  |
| Yes | 37 (94.9) |  |
|  | | |
| It was made clear what type of AAC may fit to which patients. | n (%) | Proportion of agreement (“rather yes” or “yes”):  100% (39/39)  95% CI: 91%-100% |
| No | 0 (0) |  |
| Rather no | 0 (0) |  |
| Neutral | 0 (0) |  |
| Rather yes | 9 (23.1) |  |
| Yes | 30 (76.9) |  |
|  | | |
| How would you rate the content of this learning video?  (German grading system) | n (%) | Mean value: 1.3  95% CI: 1.1-1.6 |
| Very good (1) | 30 (76.9) |  |
| Good (2) | 8 (20.5) |  |
| Satisfactory (3) | 0 (0) |  |
| Sufficient (4) | 0 (0) |  |
| Poor (5) | 0 (0) |  |
| Deficient (6) | 1 (2.6) |  |
|  | | |
| What are your suggestions for improvement regarding the content of this learning video?  (free text field) | | |
| Videos of patients using AAC; wish for more technical details | | |

Table 5: Content; AAC = augmentative and alternative communication.

**Design (Table 6)**

Students also rated the photos, the texts, and the speaker. 29 (74.4%) stated that the chosen photos made the topic clear. Here, 6 (15.4%) said “rather yes,” 3 (7.7%) “neutral,” 1 (2.6%) “no,” and none “rather no.” 31 (79.5%) found the texts comprehensible and on point (“yes”), 8 (20.5%) said “rather yes,” and none “neutral,” “rather no” or “no.” 36 (92.3%) said “yes” to “The speaker’s utterances were clear and comprehensible.” Here, 2 (5.1%) said “rather yes” and 1 (2.6%) “neutral.” When asked whether the slides’ design was appealing, 26 (66.7%) said “yes,” 11 (28.2%) “rather yes,” 2 (5.1%) “neutral,” and none “rather no” or “no.” The statement “The quality (resolution and audio) was good” was confirmed with “yes” by 34 (87.2%) of the students and with “rather yes” by 5 (12.8%). 33 (84.6%) rated the design with “very good,” which is the best grade of the German grading system. 6 (15.4%) gave the second-best grade “good.” In the free text fields, the students suggested a faster speaking tempo. In addition, it was recommended to add videos of patients using AAC and photos of patients with specific diseases.

| The selection of photos made the topic clear. | n (%) | Proportion of agreement (“rather yes” or “yes”):  89.7% (35/39)  95% CI: 75.8%-97.1% |
| --- | --- | --- |
| No | 1 (2.6) |  |
| Rather no | 0 (0) |  |
| Neutral | 3 (7.7) |  |
| Rather yes | 6 (15.4) |  |
| Yes | 29 (74.4) |  |
|  | | |
| The texts were comprehensible and on point. | n (%) | Proportion of agreement (“rather yes” or “yes”):  100% (39/39)  95% CI: 91%-100% |
| No | 0 (0) |  |
| Rather no | 0 (0) |  |
| Neutral | 0 (0) |  |
| Rather yes | 8 (20.5) |  |
| Yes | 31 (79.5) |  |
|  | | |
| The speaker’s utterances were clear and comprehensible. | n (%) | Proportion of agreement (“rather yes” or “yes”):  97.4% (38/39)  95% CI: 86.5%.99.9% |
| No | 0 (0) |  |
| Rather no | 0 (0) |  |
| Neutral | 1 (2.6) |  |
| Rather yes | 2 (5.1) |  |
| Yes | 36 (92.3) |  |
|  | | |
| The design of the slides was appealing. | n (%) | Proportion of agreement (“rather yes” or “yes”):  94.9% (37/39)  95% CI: 82.7%-99.4% |
| No | 0 (0) |  |
| Rather no | 0 (0) |  |
| Neutral | 2 (5.1) |  |
| Rather yes | 11(28.2) |  |
| Yes | 26 (66.7) |  |
|  | | |
| The quality (resolution and audio) was good. | n (%) | Proportion of agreement (“rather yes” or “yes”):  100% (39/39)  95% CI: 91%-100% |
| No | 0 (0) |  |
| Rather no | 0 (0) |  |
| Neutral | 0 (0) |  |
| Rather yes | 5 (12.8) |  |
| Yes | 34 (87.2) |  |
|  | | |
| How would you rate the design?  (German grading system) | n (%) | Mean value: 1.2  95% CI: 1-1.3 |
| Very good (1) | 33 (84.6) |  |
| Good (2) | 6 (15.4) |  |
| Satisfactory (3) | 0 (0) |  |
| Sufficient (4) | 0 (0) |  |
| Poor (5) | 0 (0) |  |
| Deficient (6) | 0 (0) |  |
|  | | |
| What are your suggestions for improvement regarding the learning video’s design?  (free text field) | | |
| Faster speaking tempo; videos of patients using AAC; more photos of patients with specific diseases | | |

Table 6: Design; AAC = augmentative and alternative communication.

**AAC in Medical Education (Table 7)**

26 (66.7%) of the 39 students said “yes” when asked “Do you find AAC important for medical doctors?” Here, 12 (30.8%) said “rather yes,” 1 (2.6%) “neutral,” and none “no” or “rather no.” Students commented that medical doctors should know how speech generating devices work and be able to communicate with patients who use AAC. Comments were also made on the lack of teaching and the need for training in AAC. When asked whether AAC should be a fixed content in their curriculum, 14 (35.9%) said “yes,” 16 (41%) “rather yes,” and 9 (23.1%) “neutral.” When asked if particularly the “***AAC***HEN”-tool (or an improved version of it) should be a fixed content in their curriculum, 14 (35.9%) said “yes,” 15 (38.5%) “rather yes,” 8 (20.5%) “neutral,” and 2 (5.1%) “rather no.” When asked how AAC should be taught (multiple answers were possible), 25 (64.1%) said “via learning videos,” 17 (43.6%) “in a lecture in presence,” 16 (41%) “via sitting in at clinical assessments (internship),” 12 (30.8%) “in an exercise seminar,” 11 (28.2%) “in an online lecture (eg, video conferencing platform like Zoom),” and 2 (5.1%) “other.” In the free text fields, students suggested to have AAC as a qualification profile (“Qualifikationsprofil” in German) or elective subject (“Wahlpflichtfach” in German) which is a voluntary, credited, specialized learning at university. The importance of face-to-face teaching was stated as well as the wish to test and explore the different types of AAC. A combination of remote and in-person teaching methods was recommended to address different learner preferences. With regards to adding the “***AAC***HEN”-tool to the RWTH toolbox-app, 20 (51.3%) said “yes,” 18 (46.2%) “rather yes,” and 1 “neutral.”

| Do you find AAC important for medical doctors? | n (%) | Proportion of agreement  (“rather yes” or “yes”):  97.4 (38/39)  95% CI: 86.5-99.9 |
| --- | --- | --- |
| No | 0 (0) |  |
| Rather no | 0 (0) |  |
| Neutral | 1 (2.6) |  |
| Rather yes | 12 (30.8) |  |
| Yes | 26 (66.7) |  |
|  | | |
| If you wish to explain your previous answer, use this free text field | | |
| Medical doctors should know how speech generating devices work; they should be able to communicate with patients who use for instance a speech generating device; common to have AAC patients (eg, with tracheotomy or Duchenne) and therefore important; lack of teaching in the study course although medical doctors often treat patients who would benefit from AAC or are AAC-users already; depends on the specialty if medical doctors need more or less knowledge, but basic knowledge is important for every doctor; more teaching necessary especially in geriatrics; indications of AAC important to know | | |
|  |  |  |
| Should AAC be a fixed content in your curriculum? | n (%) | Proportion of agreement  (“rather yes” or “yes”):  76.9 (30/39)  95% CI: 60.7-88.9 |
| No | 0 (0) |  |
| Rather no | 0 (0) |  |
| Neutral | 9 (23.1) |  |
| Rather yes | 16 (41) |  |
| Yes | 14 (35.9) |  |
|  | | |
| Should this video (or an improved version of it) be a fixed content in your curriculum? | n (%) | Proportion of agreement  (“rather yes” or “yes”):  74.4 (29/39)  95% CI: 57.9-87 |
| No | 0 (0) |  |
| Rather no | 2 (5.1) |  |
| Neutral | 8 (20.5) |  |
| Rather yes | 15 (38.5) |  |
| Yes | 14 (35.9) |  |
|  | | |
| How should AAC be taught?  (multiple choice) | n (%) | 95% CI (%) |
| Lecture in presence | 17 (43.6) | 27.8-60.4 |
| Lecture online | 11 (28.2) | 15-44.9 |
| Learning videos | 25 (64.1) | 47.2-78.8 |
| Internship (sitting in at clinical assessments) | 16 (41) | 25.6-57.9 |
| Exercise seminar | 12 (30.8) | 17-47.6 |
| Other | 2 (5.1) | .6-17.3 |
|  | | |
| If you wish to explain your previous answer, use this free text field | | |
| Learning videos are helpful, but also important to teach face-to-face for direct feedback; wish to test and explore the different types of AAC; qualification profile or elective; wish for practical experience; combination of all teaching methods to address every learner style | | |
|  | | |
| Should this video be added to our RWTH toolbox-app? | n (%) | Proportion of agreement  (“rather yes” or “yes”):  97.4% (38/39)  95% CI: 86.5%-99.9% |
| No | 0 (0) |  |
| Rather no | 0 (0) |  |
| Neutral | 1 (2.6) |  |
| Rather yes | 18 (46.2) |  |
| Yes | 20 (51.3) |  |

Table 7: AAC in medical education; AAC = augmentative and alternative communication.

|  | Knew rather well,  % (n/N) | Insufficient knowledge | | Proportion insufficient knowledge,  % (n/N) | Proportion insufficient knowledge,  95% CI,  % | |
| --- | --- | --- | --- | --- | --- | --- |
|  |  | Had an idea of,  % (n/N) | Never heard of,  % (n/N) |  |  |  |
| Heard of AAC before | 2.6  (1/39) | 59  (23/39) | 38.5  (15/39) | 97.4  (38/39) | 86.5-99.9 | |
|  | | | | | | |
|  | Memorize content better,  % (n/N) | Watch whole video,  % (n/N) | More attention to video,  % (n/N) | Did not affect me,  % (n/N) | I don’t know,  % (n/N) | |
| Announcement knowledge quiz | 53.9  (21/39) | 53.9  (21/39) | 69.2  (27/39) | 5.1  (2/39) | 0  (0/39) | |
| 95% CI,  % | 37.2-69.9 | 37.2-69.9 | 52.4-83 | 0.6-17.3 | 0-9 | |
|  | | | | | | |
|  | 100% correct,  % (n/N) | 92%  correct,  % (n/N) | 83%  correct,  % (n/N) | < 83%  correct,  % (n/N) | Mean value,  % (n/N) | 95% CI,  % |
| Knowledge quiz results | 26.5  (9/34) | 52.9  (18/34) | 20.6  (7/34) | 0  (0/34) | 92.3  (34/34) | 90.2-94.3 |

Additional tables: Students’ answers in the feedback form (N=39) and knowledge quiz results (N=34); AAC = augmentative and alternative communication.
