# Supplementary material for: “AACHEN” e-Learning Tool in Augmentative and Alternative Communication for Medical Students in Germany: Cross-Sectional Evaluation Study
Source: JMIR Med Educ. 2026 Apr 29;12:e88173. doi: 10.2196/88173 (PMC13127592; doi:10.2196/88173)
Supplement: Multimedia Appendix 6 [file mededu-v12-e88173-s006.docx]

**Interpretation**

*Prior Knowledge*

It is not surprising that most of the students were not familiar with AAC since it is only rarely or never taught in medical study courses. Sources of prior knowledge were a mixture of experiences in the students’ private life (eg, television, social media, friends, and family) and university-related events (clinical placements and internships). Some students mentioned that they heard of AAC in a vocational school or at a lecture at university. Our explanation for this is that some medical students probably already have a degree in nursing, paramedics, or speech-language pathology where AAC might have been part of the curricula.

*Knowledge Gain*

It is gratifying to note that most of the students gained knowledge from our tool. One could argue that correct answers in the knowledge quiz were due to prior knowledge or that the students simply guessed. However, the students attributed their knowledge gain to our tool. Another point is worth noting: Since this study was not monetarily compensated, the only benefit or motivation was a potential knowledge gain. One could argue that only those who perceived the “***AAC***HEN”-tool as beneficial completed the study. On the other hand, cancellation could have had various other reasons (eg, time management, technological challenges, or simply forgetting about the study, thus never initiating the study at all).

*Knowledge Quiz*

The overall perception of the knowledge quiz was positive. Students felt motivated rather than intimidated by it, which was an interesting result. This shows that it is very important to challenge students with quizzes after a lecture. In addition, quizzes should be announced prior to learning.

*Content*

For us, it was challenging to include all the important knowledge in a 25-minutes video. We are therefore delighted that the content was so positively received. We would like to expand the video and incorporate the students’ suggestions (eg, videos of patients using AAC). For this study, we kept the video to 25 minutes because we did not want to discourage the participants with a long study.

*Design*

Overall, the photos, texts, and the speaker were rated positively. Only minor improvement suggestions (eg, speaking tempo, choice of photos) were made. After a period of reflection, we would like to re-record the video for several reasons: The name of our clinic and our university’s design for presentation-slides has changed. In addition, we would like to add photos and videos. With regards to the speaking tempo, we will add a note that the video can be played at 1.5 or 2 times the speed.

*AAC in Medical Education*

We hoped that our students would see the importance of AAC and are stunned that the vast majority did. We completely understand the wish for a practical “hands on” experience in AAC, especially with regards to testing technological devices. For this study, we had to ensure a clear protocol and therefore “only” offered an e-learning tool. However, for future learning experiences, we recommend a combination of a standardized e-learning tool and “sit-in sessions” where students and their professor interact and explore different types of AAC.

**Limitations**

*Sample Size and Homogeneity*

39 is a low sample size considering that approximately 600 students were asked to participate in our study and 147 signed up for it. However, this number is sufficient for us to state a clear trend. It is possible that all 147 students watched the learning video, performed the knowledge quiz, but did not complete the feedback form. It is also possible that some students never initiated the study at all. We can only speculate, since the study was anonymous. We think that the sample size could have been much higher if we monetarily compensated the students for their time and effort. The group of students was homogenous since almost all students were in semester 6 and 7. This is because our phoniatrics lecture is scheduled at the end of semester 6. One could argue that we cannot know whether AAC might be taught in higher semesters. Thus, asking the students at the end of the study course (eg, in semester 12) could have brought different results in terms of prior knowledge.

*Semi-Objective Knowledge Evaluation*

The students shared their knowledge quiz scores in the feedback form, rather than performing an electronic test. One could argue that students might have given us wrong scores due to incorrect counting, incorrect memorization, or bad intentions. However, we think that 12 questions is a manageable amount to calculate. Also, students could type in their score immediately after the knowledge quiz, which makes memory loss unlikely. Due to the anonymous nature of the study, we do not see reasons why students would fake their scores. Also, the provision of the scores was voluntary which makes lying even more unnecessary. The guessing probability was 33%, which is high. However, students said they found the questions easy. We assume that there was no need to guess. With regards to prior knowledge, we focused on the students’ personal opinions rather than on objective scores. Due to time constraints, a preliminary test was not conducted.

*Levels of Measurement*

The scales, single-choice options, multiple-choice options, and free text fields represent different measurements. We tried our best to choose the most appropriate method for each of our objectives. However, in terms of the German grading system, we figured that it is not transferable to other grading systems used worldwide. For future international studies, this should be taken into consideration.

**Comparison to Previous Work**

To the best of our knowledge, our study is the first to test an AAC tool for medical students in Germany. Given that, our study cannot directly be compared to other studies. Other studies address the importance of including the voices of AAC users in every step of the instructional process [1,2] or show learners’ interest in first-hand experience with AAC users [3]. Our study supports this idea, since students expressed (in the free text fields) the wish to explore and experience the types of AAC in use. Many studies exist in the field of e-leaning in medicine such as the use of apps [4,5], gamification or gamified learning [6,7], or podcasts [8], to only name a few.

Furthermore, our research adds to the findings of various studies who describe the benefits of using tests and quizzes in education. Murphy et al. found that tests support learning due to retrieval practice [9], Ross et al. describe motivational factors of quizzes [10], and Salas-Morera et al. show that including quizzes has a positive effect on students’ academic performance [11].

Overall, our study contributes to the field of e-learning research in medicine and raises awareness for AAC and its significance in medical education.

**References**

1. Blasko G, Light J, McNaughton D, Williams B, Zimmerman J. Nothing about AAC users without AAC users: a call for meaningful inclusion in research, technology development, and professional training. *Augment Altern Commun*. 2025;41(3):184-194. doi:10.1080/07434618.2025.2514748
2. Balandin S, Hines M. The involvement of people with lifelong disability and communication impairment in lecturing to speech-language pathology students. Int. *J Speech-Lang Pathol*. 2011;13(5):436-445. doi:10.3109/17549507.2011.513738
3. Goldman A, Jackson D, Kannan K, et al. Training needs in augmentative and alternative communication: a virtual roundtable discussion. *Assist Technol Outcomes Benefits*. 2021;15(1):1-28.
4. Zolfaghari M, Shirzadi S, Motamed M. Using a mobile application for psychiatry training in medical students: a quasi-experimental study. *Australas Psychiatry*. 2023;31(3):389-394. doi:10.1177/10398562231159509
5. Zikos Dimitrios, Ragina Neli, Strong Oliver. Enhancing medical education with data-driven software: the TrainCoMorb app. In: *Studies in Health Technology and Informatics*. IOS Press; 2020. doi:10.3233/SHTI200499
6. Zohari M, Karim N, Malgard S, Aalaa M, Asadzandi S, Borhani S. Comparison of gamification, game-based learning, and serious games in medical education: a scientometrics analysis. *J Adv Med Educ Prof*. 2023;11(1). doi:10.30476/jamp.2022.94787.1608
7. Pérez-Baena AV, Rudolphi-Solero T, Lorenzo-Álvarez R, Ruiz-Gómez MJ, Sendra-Portero F. Gamified learning in a virtual world for undergraduate emergency radiology education: quasi-experimental study. *JMIR Med Educ*. 2025;11:e68518-e68518. doi:10.2196/68518
8. Zhang E, Trad N, Corty R, Zohrob D, Trivedi S, Rodman A. How podcasts teach: a comprehensive analysis of the didactic methods of the top hundred medical podcasts. *Med Teach*. 2022;44(10):1146-1150. doi:10.1080/0142159X.2022.2071691
9. Murphy DH, Little JL, Bjork EL. The value of using tests in education as tools for learning—not just for assessment. *Educ Psychol Rev*. 2023;35(3):89. doi:10.1007/s10648-023-09808-3
10. Ross B, Chase AM, Robbie D, Oates G, Absalom Y. Adaptive quizzes to increase motivation, engagement and learning outcomes in a first year accounting unit. *Int J Educ Technol High Educ*. 2018;15(1):30. doi:10.1186/s41239-018-0113-2
11. Salas-Morera L, Arauzo-Azofra A, García-Hernández L. Analysis of online quizzes as a teaching and assessment tool. *J Technol Sci Educ*. 2012;2(1):39-45. doi:10.3926/jotse.30
